# Improving Triethylamine-Sensing Performance of WO_3_ Nanoplates through In Situ Heterojunction Construction

**DOI:** 10.3390/s24175606

**Published:** 2024-08-29

**Authors:** Kuan Tian, Kai Yang, Xuening Ren, Yuxin Miao, Mengyao Liu, Mingxing Su, Jiawen Wu, Yu’an Sun, Pengcheng Xu

**Affiliations:** 1 Department of Material and Chemical Engineering, Zhengzhou University of Light Industry, Zhengzhou 450001, China; tiankuan@zzuli.edu.cn (K.T.); yangkai199502@163.com (K.Y.); rxningxback@163.com (X.R.); ddsmhxx@163.com (Y.M.); lmy20030111@163.com (M.L.); smx2625875903@163.com (M.S.); wjw021127@gmail.com (J.W.); 2 State Key Lab of Transducer Technology, Shanghai Institute of Microsystem and Information Technology, Chinese Academy of Sciences, Shanghai 200050, China

**Keywords:** ZnWO_4_, WO_3_, heterojunction, gas sensor, surface engineering

## Abstract

Surface engineering techniques can be used to develop high-performance gas sensing materials and advance the development of sensors. In this study, we improved the gas sensing performance of two-dimensional (2D) WO_3_ nanoplates by combining surface Zn modification and the in situ formation of ZnWO_4_/WO_3_ heterojunctions. Introducing Zn atoms by surface modification can reconstruct the atomic surface of 2D WO_3_ nanoplates, creating additional active sites. This allowed for the preparation of various types of ZnWO_4_/WO_3_ heterojunctions on the surface of the WO_3_ nanoplates, which improved the selectivity and sensitivity to the target gas triethylamine. The sensor exhibited good gas sensing performance for triethylamine even at low operating temperatures and strongly resisted humidity changes. The ZnWO_4_/WO_3_ material we prepared demonstrated a nearly threefold improvement in the triethylamine (TEA) response, with a gas sensing responsivity of 40.75 for 10 ppm of TEA at 250 °C. The sensor based on ZnWO_4_/WO_3_ has a limit of detection (LOD) for TEA of 200 ppb in practical measurements (its theoretical LOD is even as low as 31 ppb). The method of growing ZnWO_4_ on the surface of WO_3_ nanoplates using surface modification techniques to form surface heterojunctions differs from ordinary composites. The results suggest that the in situ construction of surface heterojunctions using surface engineering strategies, such as in situ modifying, is a practical approach to enhance the gas sensing properties and resistance to the humidity changes of metal oxide materials.

## 1. Introduction

Metal oxide semiconductor-based gas sensors play an important role in our lives and are an essential manifestation of technological progress and improved living standards. Currently, these sensors have been widely used in various fields, including safety measures, quality control, and personal health monitoring, where accurate gas detection using sensors is crucial. There are numerous materials with gas-sensitive properties, and the extensive literature suggests that WO_3_ is an effective gas-sensitive material [1,2]. However, to better meet the application of gas sensors based on WO_3_ in real life, it is necessary to further enhance the gas sensing performance of WO_3_, such as low sensitivity, poor selectivity, and poor humidity immunity [3]. Various strategies have been implemented in previous studies to enhance the gas sensing properties of WO_3_ [2,4,5,6,7]. Surface structure plays a crucial role in improving gas sensing performance, as the conduction behavior of metal oxides is influenced by surface chemical adsorption, reactions, and catalysis during gas molecule interaction [3]. It is conceivable that surface modification of metal oxides can substantially improve their gas sensing capabilities [8].

**Figure 1:**
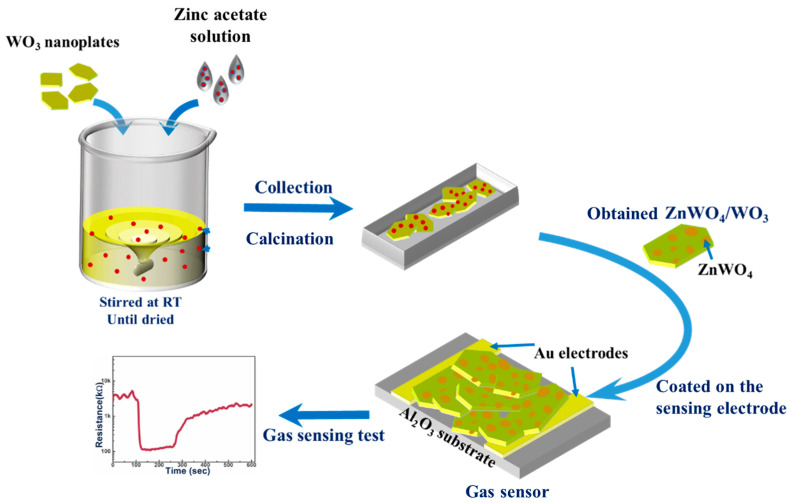
Schematic diagram illustrating the in-situ construction of a heterojunction on WO_3_ nanoplates and the preparation of gas sensors.

In this study, we introduced Zn^2+^ ions through rational modifying into the surface of WO_3_ nanoplates, forming a unique internal surface heterojunction composed of ZnWO_4_/WO_3_ (as illustrated in Figure 1). This heterojunction structure is akin to heteroatom-doped metal oxides but confined explicitly to the surface, generating shallow energy levels within the base material and inducing rearrangements in the atomic structure. The internal heterojunction formed within the substrate oxide effectively segregates electron-hole pairs. Consequently, the ZnWO_4_/WO_3_ internal heterojunction structure has been extensively employed in photocatalysis and optoelectronic semiconductor applications [9,10]. Leveraging the efficient charge separation capability of this internal heterojunction, the ZnWO_4_/WO_3_ heterojunction improves the adsorption and reaction of target gases on the surface of WO_3_. Moreover, the incorporation of ZnWO_4_/WO_3_ heterojunctions into the surface of WO_3_ nanoplates enhances the utilization of composites for H_2_O in the air, promoting the production of more active radicals on the surface while reducing the impact of humidity on gas sensing performance and enhancing the response of the composites [11].

To explore the preparation process of this distinctive heterojunction and its influence on the gas sensing performance of WO_3_, we synthesized various ZnWO_4_/WO_3_ nanoplates with internal heterojunctions using an ion permeability method. The gas sensing performance of the sensors based on ZnWO_4_/WO_3_ nanoplates exhibited significant improvement. Notably, the gas sensing response to triethylamine (TEA) at low operating temperatures exhibited a remarkable increase, approximately four times higher than the response of WO_3_ to 10 ppm TEA at 250 °C (R_a_/R_g_ = 40.75). TEA is a colorless and transparent toxic gas with a strong ammonia odor, widely used in industrial and commercial applications. According to the Occupational Safety and Health Administration (OSHA) suggestion, the threshold limit value (TLV) for TEA is 10 ppm [12], and the American Conference of Governmental Industrial Hygienists (ACGIH) recommends a TLV of 1 ppm [13]. Fast, sensitive, and selective TEA detection is crucial for the chemical industry and our everyday lives.

Structural characterization using XRD, TEM, and EDS revealed that the ZnWO_4_ phase is embedded within the WO_3_ nanoplates, forming “nano-dots”, “nano-islands”, or “continuous domain” structures on the surface. This island-like distribution of the ZnWO_4_/WO_3_ heterojunctions, coupled with efficient electron-hole separation, results in electron-rich regions on the surface of the WO_3_ nanoplates. This alteration affects the adsorption and reaction of target gases on the surface of WO_3_, consequently strongly influencing the electrical properties of WO_3_. The findings of this study demonstrate that the internal heterojunction of ZnWO_4_/WO_3_ constructed on the surface of nanoplates plays a crucial role in enhancing the gas sensing performance of WO_3_. In comparison to uniformly distributed heterojunctions and additive heterojunctions, the surface internal heterojunction structure presented in this study, achieved through a combination of surface engineering and band structure design technology, offers innovative ideas and methodologies for fabricating high-performance gas sensors based on metal oxides and advancing the development of novel gas sensing materials.

## 2. Experimental Section

### 2.1. Material Synthesis

All reagents were of analytical-reagent grade and used as purchased from Sinopharm Chemical Reagent Co. Ltd. (Shanghai, China). without any purification. The preparation procedures for the WO_3_ nanoplates and the ZnWO_4_/WO_3_ nanoplates are demonstrated below.

### 2.2. Preparation of WO_3_ Nanoplates

The WO_3_ nanoplates were prepared using a low-temperature method. The typical process is that 3.3 g Na_2_WO_4_·2H_2_O was dissolved into 50 mL deionized water with stirring. Next, 10 mL hydrochloric acid (36–38%) was added. Then, 0.9 g oxalic acid was added under stirring and aging at 60 °C for 24 h. Finally, the precursor was washed and centrifuged. After drying at 60 °C for 12 h, the precursor was calcined at 450 °C with a rate of 1 °C/min for 2 h, and the WO_3_ nanoplates were obtained.

### 2.3. Preparation of Nanoplates with ZnWO_4_/WO_3_ Surface Heterojunction

The WO_3_ nanoplates prepared above were used as a substrate for ZnWO_4_/WO_3_. First, 0.46 g WO_3_ nanoplates (0.002 mol) were impregnated in a 10 mL solution with different concentrations of zinc acetate (0.01 M, 0.1 M, and 0.5 M) with ultrasonic dispersion for 10 min. After that, the suspension solutions were subsequently stirred at room temperature until dried. The obtained samples were annealed at 500 °C with a rate of 1 °C/min for 2 h. The as-prepared samples were labeled as ZnWO_4_/WO_3_-0.01, ZnWO_4_/WO_3_-0.1, and ZnWO_4_/WO_3_-0.5.

### 2.4. Microscopic Characterization

X-ray diffraction (XRD) patterns of these samples were recorded by a Rigaku D/Max-2500 X-ray diffractometer (Rigaku Corporation, Tokyo, Japan) using Cu-Kα1 as radiation. High-resolution transmission electron microscopy (HR-TEM) analyses and energy dispersive X-ray (EDX) mapping profiles were conducted on an FEI Talos F200X G2 microscope (Thermo Scientific, Waltham, MA, USA) with energy dispersive spectroscope (super-x, Energy resolution < 136 eV (Mn-Ka)) at an accelerating voltage of 200 kV. X-ray photoelectron spectroscopy (XPS) was performed using an ESCA LAB250Xi spectrometer (Thermo Scientific, USA) with an unmonochromated Al Kα X-ray source. The energy resolution of XPS is less than or equal to 0.45 eV. The UV–vis spectra were recorded on a Thermo Scientific Evolution 201 spectrophotometer (Thermo Scientific, USA). The Raman spectra were recorded with a 532 nm wavelength laser using a HORIBA XploRA PLUS.3 (HORIBA, Longjumeau, France).

### 2.5. Gas Sensing Measurement

The measurement of gas-sensing properties was similar to that of reported work [14,15]. The gas sensing performance was evaluated using the WS-30A test instrument (Hanwei Electronics Co. Ltd., Zhengzhou, China). Target gases of various concentrations were obtained by heating a specific amount of analytically pure liquid chemicals to generate vapor, which was then mixed with clean air. The test chamber we used in this work has a volume of 18 L. According to the ideal gas law, the vapor concentration can be calculated based on the quantity of the liquid sample injected. For example, to achieve the desired concentration of triethylamine or other vapors, precise volumes of solution are injected into the hot plate of the test chamber to ensure complete evaporation. Simultaneously, the fan in the test chamber was activated to ensure an even distribution of the target gas throughout the chamber. During testing, we maintained the relative humidity of the sample chamber at approximately the same level as the laboratory environment, within the range of 35 ± 5%.

After exposing the gas sensor to the sample gas environment for 180 s, we opened the test chamber and used a fan to blow away the sample gas. This restored the test environment to the initial clean air. We then prepared at least 4 sensors of each type and tested each sensor more than 5 times to obtain the corresponding error bars. The response of these sensors is defined as S = R_g_/R_a_, where R_a_ is the average resistance of the sensor in the air, and R_g_ is the average resistance of the sensor in the target gas. The time it takes for the sensor to reach 90% of the complete response and recovery is called the response time (t_res_) or recovery time (t_rec_). The aging and testing processes were conducted in a separate room. The humidity of the gas mixture was controlled with a saturated inorganic salt solution. Relative humidity of 33%, 43%, 52%, 67%, 75%, and 85% were prepared by using MgCl_2_, K_2_CO_3_, Mg(NO_3_)_2_, CuCl_2_, NaCl, and KCl saturated solutions, respectively.

**Figure 2:**
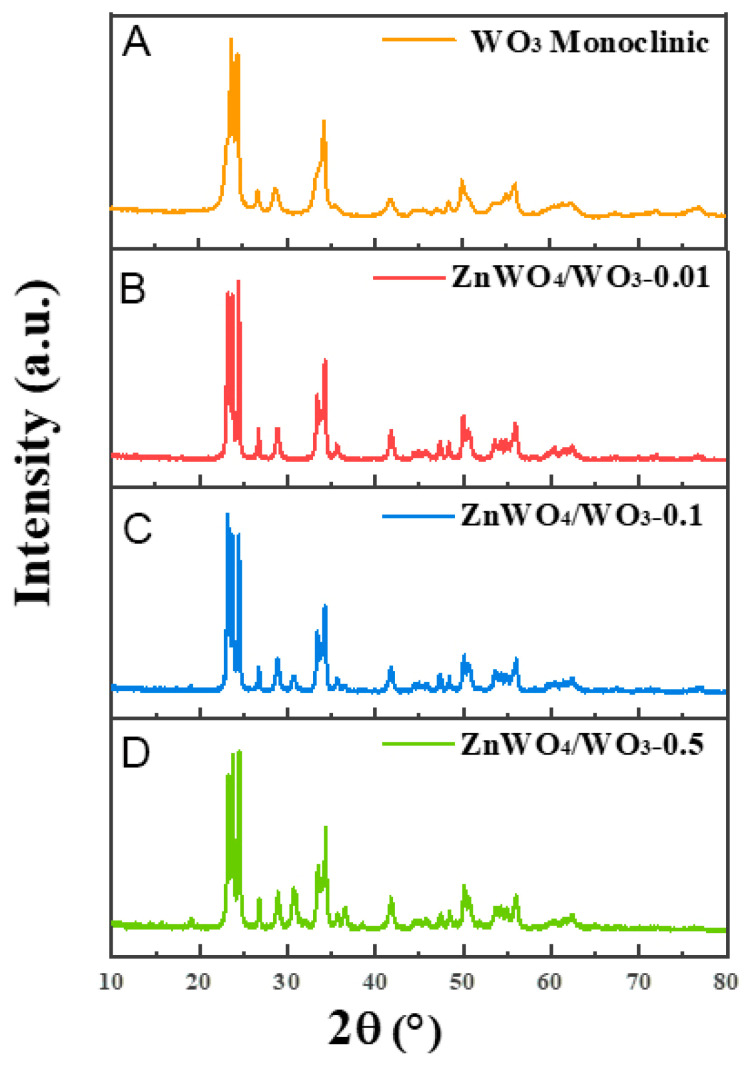
XRD patterns of (**A**) WO_3_, (**B**) ZnWO_4_/WO_3_-0.01, (**C**) ZnWO_4_/3-0.1, and (**D**) ZnWO_4/_WO_3_-0.5.

As investigated in Figure 2, the XRD patterns of the WO_3_ nanoplates and these different ZnWO_4_/WO_3_ samples are clearly displayed. The strong diffraction peaks in the XRD pattern of WO_3_ nanoplates can be well-indexed to WO_3_ (JCPDS NO.72-0677). It can be observed from spectra in Figure 2B–D that the intensity of the ZnWO_4_ (100), (1–11), and (021) crystal planes increase gradually with the increasing concentration of Zn^2+^. And these peaks indicate precisely the type of monoclinic structure (JCPDS NO.73-0554). For the content of ZnWO_4_, which is far below the limit of detection of XRD, no prominent peaks of the ZnWO_4_ phase are observed in the spectrum of ZnWO_4_/WO_3_-0.01 (as shown in Figure 2B). It is considered that the doped Zn^2+^ is too small to form a continuous ZnWO_4_ phase on the surface of WO_3_ nanoplates, and Zn^2+^ modifying into the WO_3_ lattice will replace the W^6+^ to create more oxygen vacancies. The results of XPS support this hypothesis. This result demonstrates the possibility of the formation of a phase.

**Figure 3:**
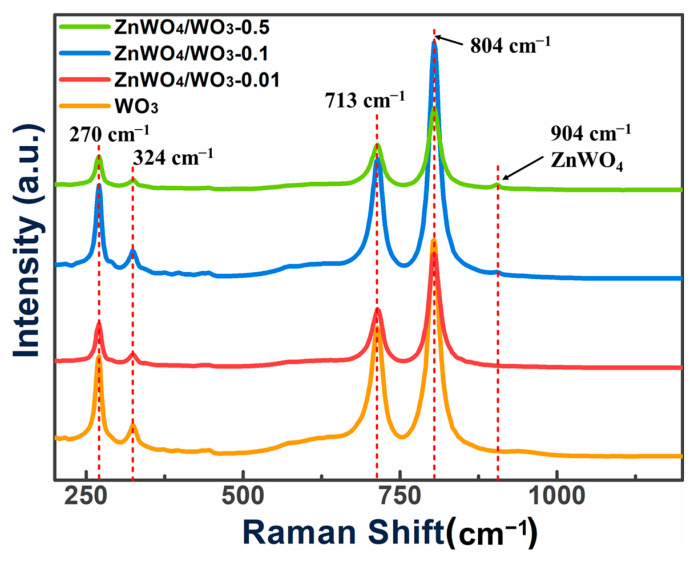
Raman patterns of the prepared four samples.

The Raman spectra of the WO_3_ nanoplates and ZnWO_4_/WO_3_ samples are shown in Figure 3 to illustrate the phase information further. It can be found that two intense peaks at 713 and 804 cm^−1^ can be clearly observed, which are mainly attributed to the monoclinic WO_3_, which is in good accordance with the XRD [16]. As depicted from the patterns of different ZnWO_4_/WO_3_ samples, a distinct peak at 904 cm^−1^ presents in the patterns of ZnWO_4_/WO_3_-0.1 and ZnWO_4_/WO_3_-0.5. This peak is ascribed to the stretching vibration of the W-O bonds of ZnWO_4_ [17]. The Raman results demonstrate that we have successfully prepared the ZnWO_4_/WO_3_ composites.

**Figure 4:**
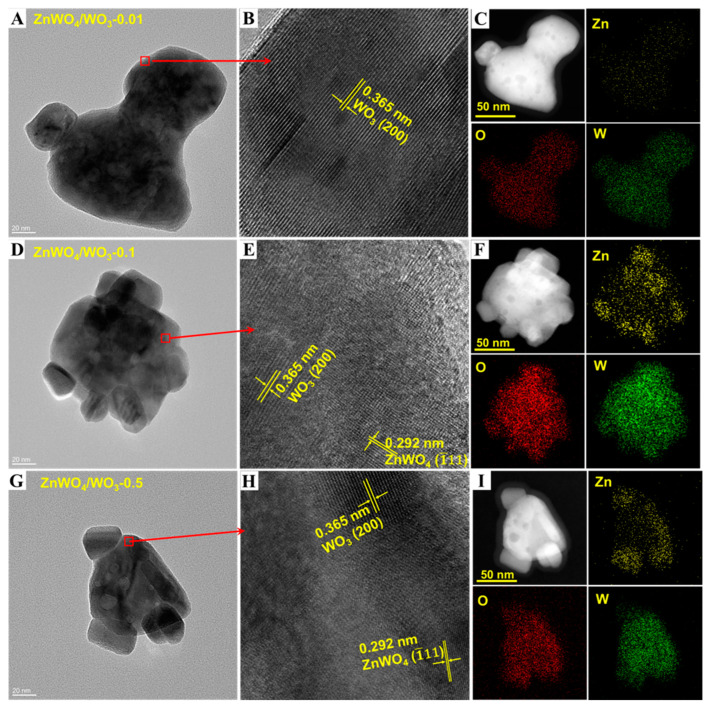
TEM images and EDS mapping patterns of the prepared ZnWO_4_/WO_3_ nanoplates. (**A**) low-magnification image of the ZnWO_4_/WO_3_-0.01 sample. The scale bar is 20 nm; (**B**) the high-magnification image of the ZnWO_4_/WO_3_-0.01 sample; (**C**) EDS mapping patterns of the ZnWO_4_/WO_3_-0.01 sample; (**D**) low-magnification image of the ZnWO_4_/WO_3_-0.1 sample. The scale bar is 20 nm; (**E**) high-magnification image of the ZnWO_4_/WO_3_-0.1 sample; (**F**) EDS elements mappings of the ZnWO_4_/WO_3_-0.1 sample, (**G**) low-magnification image of the ZnWO_4_/WO_3_-0.5 sample. The scale bar is 20 nm; (**H**) high-magnification image of the ZnWO_4_/3-0.5 sample; (**I**) EDS mapping results of the ZnWO_4_/WO_3_-0.5 sample.

TEM was used to study the surface morphology and structural characteristics of ZnWO_4_/WO_3_, as shown in Figure 4. It can be observed that WO_3_ forms a nanoplate structure with an average size of about 150 × 80 nm, and the size distribution of the WO_3_ nanoplates is uniform. The substrate WO_3_ nanoplates’ structure is well preserved after the in situ construction of the ZnWO_4_/WO_3_ composites. It precisely shows that the ZnWO_4_/WO_3_ composites retain the uniform nanoplates’ morphology of the substrate, and the surface of the ZnWO_4_/WO_3_ composites is as smooth as the WO_3_ nanoplates. This result indicates that these ZnWO_4_/WO_3_ heterojunction composites are not composed of uniform separated ZnWO_4_ and WO_3_ phases, but a new phase ZnWO_4_ region has been formed on the surface of the WO_3_ nanoplates through the Zn^2+^ ions that have penetrated the WO_3_ crystal lattice.

Furthermore, no new structures are found in these composites, which indicates that the ZnWO_4_ phase may be embedded into the surface of WO_3_ nanoplates. Hence, the improvement in the gas sensing performance of these composites is mainly brought by the change in the surface structure of the WO_3_ nanoplates. The lattice fringes can be observed in Figure 4B,E,H. The spacing of 0.365 nm can be indexed to the (200) plane of monoclinic WO_3_, while the other spacing of 0.292 nm matches the (1–11) plane of ZnWO_4_. It should be noted that no apparent lattice can be found in ZnWO_4_/WO_3_-0.01. This is probably attributed to the size of the ZnWO_4_ phase being tiny. The high-angle annular dark field (HAADF) elemental mapping images (Figure 4C,F,I) can be applied to verify the existence of ZnWO_4_ phase on the surface of the WO_3_ nanoplates. It is clearly investigated that the Zn elements are evenly dispersed on the surface of the WO_3_ nanoplates, and the dispersed concentration of Zn elements will increase with the increase in the impregnating solution concentration of Zn^2+^. As shown in the EDS mapping results of ZnWO_4_/WO_3_-0.01 (Figure 4C), it can be observed that a small amount of the Zn element is scattered on the surface of the WO_3_ nanoplates. This result indicates that some Zn^2+^ penetrates the lattice of WO_3_ to form ZnWO_4_, and the amount of ZnWO_4_ distributed as dots on the surface of WO_3_ nanoplates is too low to be detected by XRD and Raman spectroscopy. As shown in Figure 4F,I, the ZnWO_4_ has a continuous phase on the surface of WO_3_ nanoplates, which looks like an “island”.

**Figure 5:**
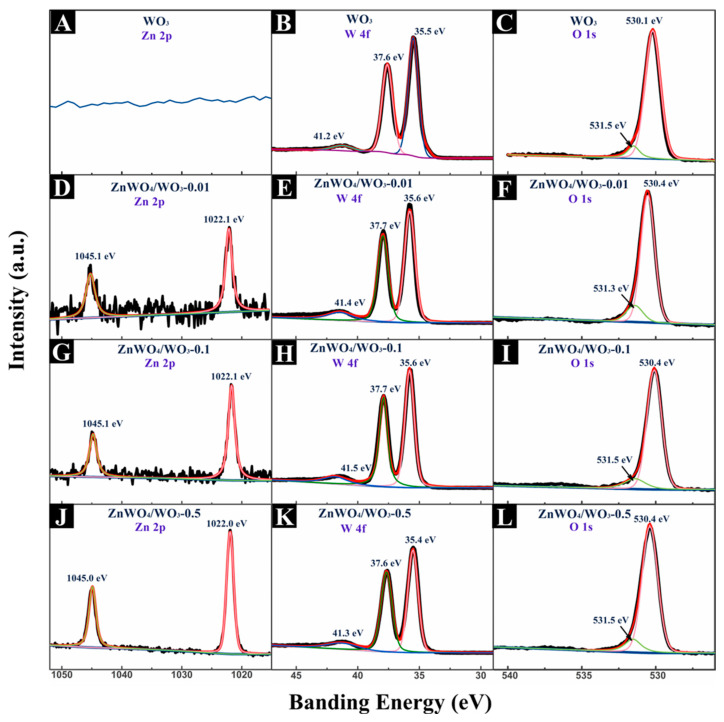
XPS spectra of the samples: (**A**–**C**) Zn 2p, W 4f and O 1s level of WO_3_ nanoplates, (**D**–**F**) Zn 2p, W 4f and O 1s level of ZnWO_4_/WO_3_-0.01 nanoplates, (**G**–**I**) Zn 2p, W 4f and O 1s level of ZnWO_4_/WO_3_-0.1 nanoplates, (**J**–**L**) Zn 2p, W 4f, and O 1s level of ZnWO_4_/WO_3_-0.5 nanoplates.

In order to investigate the definite elemental constituent and chemical states of the ZnWO_4_/WO_3_ internal heterojunction, XPS analyses were adopted in this work. The XPS data were calibrated using the 284.4 eV peak of the C element as the standard, employing the Tougaard method to conduct background correction on all spectra, and then utilizing the Lorentz–Gaussian function for peak fitting to determine the corresponding fitted peaks. Figure 5 investigates the high-resolution XPS spectra of Zn 2p, W 4f, and O 1s. The high-resolution Zn 2p spectra of all the ZnWO_4_/WO_3_ nanoplates exhibited two distinct spin-orbit doublet peaks located at about 1045 and 1022 eV, which verified that the Zn element holds an oxidation state of +2 in the composites [9,18]. The W 4f spectra can be observed in Figure 5B,E,H,K. Three peaks were clearly observed after fitting with the Gaussian–Lorentzian functions in all samples. The two characteristic peaks of W 4f_5/2_ and W 4f_7/2_ at binding energies of 37.7 eV and 35.5 eV are typical for W^6+^ sites coordinated by O^2−^ [19,20,21]. The O 1s spectra of all samples are shown in Figure 5C,F,I,L. The O 1s region of WO_3_ and ZnWO_4_/WO_3_ nanoplates can be fitted by two peaks corresponding to the lattice oxygen and oxygen vacancy [9,20]. These results indicate that the chemical states of the W and O elements are the same in all samples, and the chemical states of the Zn element are not significantly different in the three different ZnWO_4_/WO_3_ nanoplates. All these features indicate that the samples are ZnWO_4_ and WO_3_, as reported in previous work [22,23].

The results of the XRD, Raman spectrum, EDS mapping, and XPS characterization indicate that Zn^2+^ has been successfully incorporated into the WO_3_ surface lattice, forming a new ZnWO_4_ phase in the microregion of the WO_3_ surface. Unlike traditional methods of metal oxide doping, our approach involves immersing WO_3_ nanosheets in a solution containing a specific concentration of Zn^2+^, thoroughly stirring to adsorb Zn^2+^ onto the surface of the WO_3_ nanosheets, and then treating them at high temperature to allow Zn^2+^ to enter the WO_3_ lattice. As a result, the doped Zn^2+^ gradually penetrates the lattice from the surface of the WO_3_ nanosheets under high-temperature conditions, becoming concentrated in the surface lattice area. Depending on the concentration of doped Zn^2+^, this process can lead to a continuous or discontinuous new ZnWO_4_ phase on the surface of the WO_3_ nanoflakes. This novel impregnation method results in the preparation of a ZnWO_4_/WO_3_ composite material with a unique surface heterojunction structure, which was added prior to Section 4 on gas sensing properties.

## 3. Gas Sensing Properties

**Figure 6:**
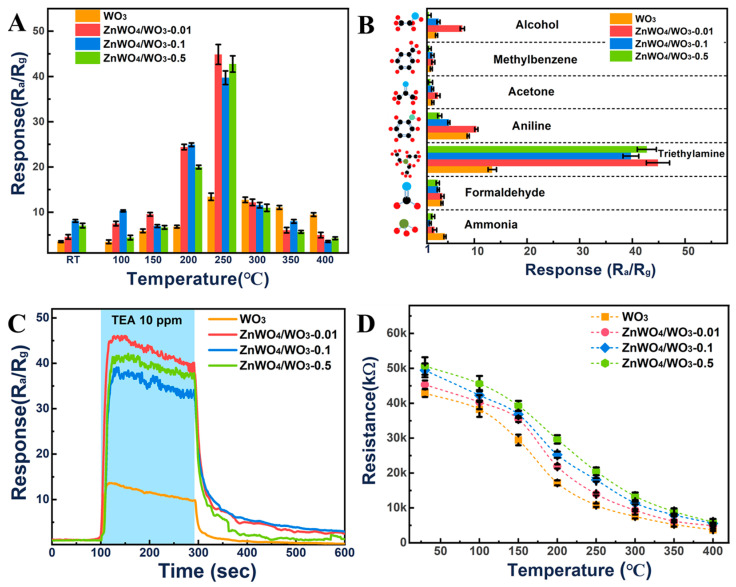
(**A**) Response of WO_3_ nanoplates, ZnWO_4_/WO_3_-0.01 nanoplates, ZnWO_4_/WO_3_-0.1 nanoplates and ZnWO_4_/WO_3_-0.5 nanoplates sensors to 10 ppm TEA at different operating temperatures, (**B**) Selectivity of WO_3_ nanoplates, ZnWO_4_/WO_3_-0.01 nanoplates, ZnWO_4_/WO_3_-0.1 nanoplates and ZnWO_4_/WO_3_-0.5 nanoplates sensors to 10 ppm of various gases at 250 °C, (**C**) Response–recovery curves of WO_3_ nanoplates, ZnWO_4_/WO_3_-0.01 nanoplates, ZnWO_4_/WO_3_-0.1 nanoplates and ZnWO_4_/WO_3_-0.5 nanoplates sensors to 10 ppm TEA at 250 °C, (**D**) the relationship between the resistance value (R_a_) of the four sensors in air and the operating temperature.

**Figure 7:**
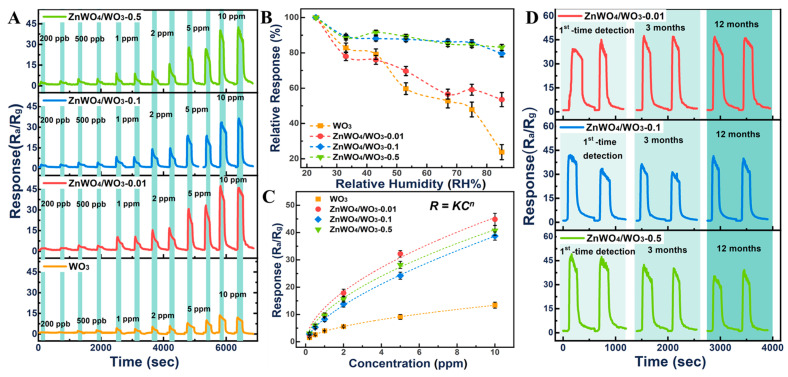
(**A**) Response–recovery curves of WO_3_ and ZnWO_4_/WO_3_ sensors to different concentrations (0.2, 0.5, 1, 2, 5 and 10 ppm) of TEA at 250 °C and the RH = 35% ± 5% (this humidity here is the ambient humidity at room temperature), (**B**) Humidity stability of different ZnWO_4_/WO_3_ sensors at 250 °C to 10 ppm TEA (the relative humidity were prepared by different saturated solution at 25 °C), (**C**) The Freundlich fitting of the sensing response to TEA concentration, (**D**) Long-term stability of ZnWO_4_/WO_3_ sensors 10 ppm TEA at 250 °C and the RH = 35% ± 5% (this humidity here is the ambient humidity at room temperature).

The sensors prepared with different samples were measured by their gas sensing performance to other volatile organic compounds (VOCs) at various concentrations (0.2–10 ppm) and operating temperatures (100–400 °C). The gas sensing performance of the WO_3_ and ZnWO_4_/WO_3_ nanoplates with varying concentrations of Zn ions are shown in Figure 6 and Figure 7. The gas sensors based on these nanoplates present the typical n-type semiconductor behavior toward TEA under an indoor humidity of about 35 ± 5%. Figure 6A exhibits the temperature-dependent response of all the gas sensors to 10 ppm TEA, with the working temperature from room temperature (RT) to 400 °C. It was found that the response of the sensors based on the different WO_3_ and ZnWO_4_/WO_3_ nanoplates increased with the increasing temperature, from RT to 250 °C. However, the response of all the sensors gradually decreased with further increases in the operating temperature. From then on, 250 °C was selected as the optimum working temperature for the gas sensing performance test. As shown in Figure 6C, the response of WO_3_ sensors is about 13.41 to 10 ppm TEA at 250 °C, and the response/recovery time is about 17/72 s. The response of the ZnWO_4_/WO_3_ sensors has been obviously improved, reaching about 44.86 (ZnWO_4_/WO_3_-0.01), 38.72 (ZnWO_4_/WO_3_-0.1), and 40.75 (ZnWO_4_/WO_3_-0.5), and the speed of response was also enhanced by the construction of surface heterojunction. The response time shortened from 17 s to 10 s as the concentration of Zn^2+^ content increased. Furthermore, it can also be found that the response of ZnWO_4_/WO_3_ nanoplates to TEA is improved at 250 °C. The selectivity of these different kinds of sensors to 10 ppm of various VOC gases at the optimum temperature of 250 °C is presented in Figure 6B. The ZnWO_4_/WO_3_ sensors exhibit good selectivity to TEA at optimum temperature. Significantly, the response of ZnWO_4_/WO_3_-0.5 sensors reaches about 40.75 towards TEA, while it only reaches 1.4–3.5 towards other VOC gases.

To investigate the repeatability and sensitivity performance of the WO_3_ and ZnWO_4_/WO_3_ nanoplates to TEA, the response–recovery curves of these four kinds of sensors’ exposure to various concentrations of TEA (0.2, 0.5, 1, 2, 5, and 10 ppm) at the optimum temperature are illustrated in Figure 7A. It can be observed that each concentration point has been tested twice, and the responses of these sensors increased with the increase in the TEA concentration. This result indicates that these sensors have good repeatability among individual alternating tests. The response of the ZnWO_4_/WO_3_-0.5 sensors gradually increases from 3.62 at 200 ppb to 40.75 at 10 ppm. Even as the concentration of TEA is 200 ppb, the response can reach about 3.62, which is two times higher than that of WO_3_, and the response–recovery time is 24/47 s. Although the response of ZnWO_4_/WO_3_-0.01 and ZnWO_4_/WO_3_-0.1 is very good, the recovery time of ZnWO_4_/WO_3_-0.01 and ZnWO_4_/WO_3_-0.1 increases more than 100 s. For verifying the effect of the surface heterojunction on the humidity immunity, the humidity-dependent testing of the WO_3_ and ZnWO_4_/WO_3_ sensors to 10 ppm TEA was measured under different RH conditions (Figure 7B). It can be found that the response values of the pure WO_3_ and ZnWO_4_/WO_3_-0.01 nanoplates dramatically decrease with the increasing RH. This is mainly due to the lesser amount of doped Zn^2+^ on the WO_3_ surface, and the formation of a new phase is not enough to offset the effect of water molecules. With the increase in the content of the ZnWO_4_ phase disturbed on the surface, the humidity immunity of the composites is significantly improved. The results revealed that at a relative humidity of 85%, the response of the sensor based on ZnWO_4_/WO_3_-0.5 was only 16% lower than that of the device at a relative humidity of 25%, indicating nice humidity immunity of the sensors based on ZnWO_4_/WO_3_-0.5. The influence of moisture on the gas sensing performance of metal oxides is mainly because the water molecules can further react with the negative oxygen ions formed on the surface of metal oxides to produce hydroxyl and directly change the resistance of the sensors. However, ZnWO_4_, as previous works have reported [23,24], presented a strong adsorption and decomposition ability to water molecules, which mainly depends on the generated reaction of the holes with the molecular water. As in the previous reports, the WO_3_ will transfer holes to the valence band of ZnWO_4_ in the ZnWO_4_/WO_3_ heterojunctions [21,25]. The ZnWO_4_ phase disturbed on the surface of the WO_3_ nanoplates will preferentially adsorb and consume a lot of water molecules. Meanwhile, the electrons of the WO_3_ surface were increased to form more negative oxygen ions. The above results offset the influence of humidity on the gas sensing performance of composites. In addition, Figure 7C illustrates the responses *R* of these sensors exhibiting a Freundlich isotherm relationship with TEA concentration *C*. After fitting with the Freundlich equation as follows:

$$R={KC}^{n}$$

the relevant parameters are presented in Table 1. The detection limit of the ZnWO_4_/WO_3_-0.5 sensors was estimated to be 1.40 toward 31 ppb of TEA as the S/N = 3. The long-term stability and reproducibility have also been characterized to evaluate the Zn^2+^ doped WO_3_ nanoplates. As shown in Figure 7D, the response/recovery curves of these ZnWO_4_/WO_3_ composites toward 10 ppm TEA at 250 °C revealed that the long-term stability and reproducibility of these ZnWO_4_/WO_3_ sensors are very good. The response value deviation of each sensor based on ZnWO_4_/WO_3_ is less than 2.2, indicating a very low drift deviation of the device. The fundamental resistance drift of each sensor at 250 °C remains below 5%, indicating its reliability in applications. This good stability and reproducibility are mainly ascribed to the composites having uniform nanoplates structural without any hierarchical nanostructure, and the WO_3_ and ZnWO_4_ have excellent thermal and chemical stability. As shown in Table 2, the obtained results demonstrated that the sensor based on ZnWO_4_/WO_3_ heterojunctions is very promising for practical application.

## 4. Gas Sensing Mechanism

**Figure 8:**
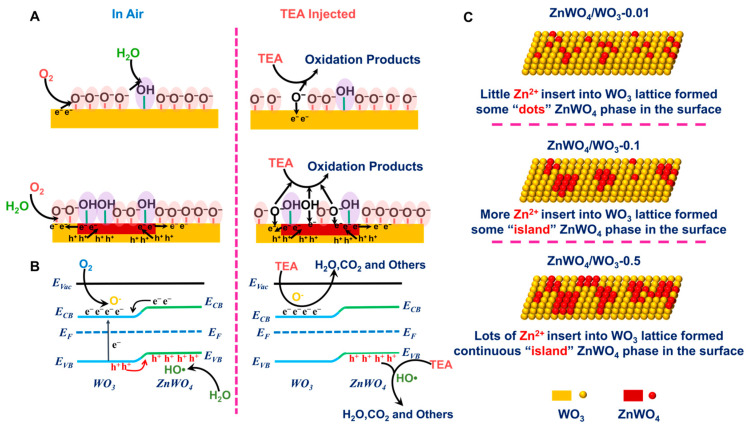
(**A**,**B**) Schematic diagram showing the sensing mechanism; (**C**) Schematic of ZnWO_4_/WO_3_ internal heterojunction nanoplates.

As described above, the ZnWO_4_ phase disturbed on the surface of the WO_3_ nanoplates can adsorb a large number of O_2_ and H_2_O to activate them and produce active radicals that participate in the gas sensing reaction (as shown in Figure 8A) [23,38]. Furthermore, more oxygen vacancies have been created during the impregnation process. Compared to ZnWO_4_/WO_3_-0.1 and ZnWO_4_/WO_3_-0.5, the gas sensing performance of ZnWO_4_/WO_3_-0.01 is mainly improved by a higher concentration of oxygen vacancy. This is probably because of the small amount of Zn^2+^, which cannot construct a continuous ZnWO_4_ phase after infiltration into the WO_3_ lattice, resulting in the local substitution of W^6+^ for producing more oxygen vacancies on the surface. Therefore, the humidity immunity of ZnWO_4_/WO_3_-0.01 is still poor. Apart from these favorable factors for enhancing the gas sensing performance of these composites, the internal heterojunctions formed on the surface of the WO_3_ nanoplates can efficiently separate the electron hole in the ZnWO_4_/WO_3_, resulting in the increase in electrons in the conduction band of WO_3_ to generate more negative oxygen ions, and the valence band of ZnWO_4_ will then attract more holes from WO_3_, which further forms active radicals (as shown in Figure 8B). This process can further improve the gas sensing performance of WO_3_ nanoplates toward TEA. Obviously, the gas sensing performance of these internal heterojunction composites did not increase geometrically with the increase in ZnWO_4_ content on the surface of the WO_3_ nanoplates. The first reason is the homogeneity of the ZnWO_4_/WO_3_ internal heterojunctions prepared by the impregnation method. It is impossible to guarantee that the ZnWO_4_ phase content and distribution on the surface of each WO_3_ nanoplate will be the same (as shown in Figure 8C). Secondly, the more the ZnWO_4_ phase is distributed on the surface, the smaller the area of WO_3_ which will participate in the gas sensing reaction, which makes the response of ZnWO_4_/WO_3_-0.5 to TEA unable to be further improved. Therefore, the rational design of the content and distribution of ZnWO_4_ on the surface of the WO_3_ nanoplates is essential for enhancing gas sensing performance. In this work, the ZnWO_4_/WO_3_-0.1 nanoplates, which demonstrate good response, selectivity, and long-term stability gas sensing performance towards TEA and fine humidity immunity, are sufficient to be applied as a gas sensing material for TEA testing in practical application. Excessive doping of Zn^2+^ on the surface of the WO_3_ nanosheets cannot further improve the gas sensing performance of TEA but will cause the unnecessary waste of the zinc element.

## 5. Conclusions

In this work, we prepared various surface-intra-heterojunction nanostructured ZnWO_4_/WO_3_ composites using a controlled impregnation method. We found that using a double impregnation method effectively created heterojunction structures on the surface of WO_3_ nanoplates. These surface heterojunction composites significantly improved gas sensing performance, particularly for TEA, through surface engineering. The ZnWO_4_/WO_3_-0.5 composite showed a nearly three times increased response and improved selectivity. It exhibited a gas sensing response of 40.75 at 250 °C to 10 ppm TEA, with a response recovery time of 10/89 s, faster than WO_3_ nanoplates. The sensor based on ZnWO_4_/WO_3_-0.5 achieved a minimum detection concentration of 200 ppb, with a response of about 3.62, which was higher than the response to other VOC gases (about 1.4~3.5). The theoretical LOD was approximately 31 ppb. The sensor based on ZnWO_4_/WO_3_-0.5 exhibited ample long-term stability. Even after 12 months of placement, the response of the sensor decreased by only 2.2. Additionally, this sensor demonstrated good humidity resistance, with the sensor response value decreasing by less than 16%, even at a relative humidity of up to 85%. These results demonstrated that sensors based on ZnWO_4_/WO_3_-0.5 present good stability and a low failure rate. Our results demonstrate the effectiveness of surface engineering in designing and optimizing the surface structures of metal oxides, especially for enhancing gas sensing performance. Our research provides a promising strategy for improving the gas sensing performance of oxide materials.

## Figures and Tables

**Table 1:** Parameters were obtained by fitting using the Freundlich equation.

Sensor	R^2^	K	*n*
WO_3_	0.9998	3.830	0.543
ZnWO_4_/WO_3_-0.01	0.9997	8.491	0.761
ZnWO_4_/WO_3_-0.1	0.9999	7.957	0.693
ZnWO_4_/WO_3_-0.5	0.9999	10.651	0.586

**Table 2:** Comparison of the gas sensing performance of various WO_3_-based sensors toward TEA.

Material	Operation Condition	Concentration (ppm)	Response	t_res_/t_rec_ (s)	LOD (ppb)
Hierarchical CPDs/WO_3_ [26]	140 °C	40	14.6	267/340	46
0.8% rGO-WO_3_-ZnFe_2_O_4_ [27]	130 °C	10	26.92	51/144	20
Hierarchical WO_3_ Flower-like Spheres [28]	205 °C	10	11.6	3/55	83
Micro-flower WO_3_ [5]	325 °C	1	2.2	3/5	50
Carbon Modified WO_3_-W_18_O_49_ with PdO [29]	325 °C	100	35.7	1/2	50
WO_3_/h-BN-5wt% [30]	260 °C	500	390.6	8/60	41
Needle-Shaped WO_3_ [31]	250 °C	1	6.4	45/78	1000
WO_3_ Hollow Microspheres [32]	220 °C	60	16	1.5/22	50,000
Hierarchical Bi_2_O_3_/WO_3_ [33]	140 °C	50	9.2	89/162	32
Co&Ni Co-Doped W_18_O_49_ Nanourchins [34]	250 °C	50	154	16/13	1000
Layered Pt/PtO_2_-WO_3_ [35]	137.5 °C	50	3323.5	16/262	100
WO_3_/WS_2_ [36]	240 °C	50	21.81	−/−	3000
In-doped WO_3_ cubic nanoblocks [37]	115 °C	50	11.2	11/40	1000
ZnWO_4_/WO_3_-0.01 (This work)	250 °C	10	44.86	16/122	93
		0.2	2.41	32/188	
ZnWO_4_/WO_3_-0.1 (This work)	250 °C	10	38.72	13/113	81
		0.2	2.89	26/178	
ZnWO_4_/WO_3_-0.5 (This work)	250 °C	10	40.75	10/89	31
		0.2	3.62	24/47	

## Data Availability

The data presented in this study are available on request from the corresponding author.
